# Oral Complications in Hematopoietic Stem Cell Recipients: The Role of Inflammation

**DOI:** 10.1155/2014/378281

**Published:** 2014-04-10

**Authors:** T. M. Haverman, J. E. Raber-Durlacher, W. M. H. Rademacher, S. Vokurka, J. B. Epstein, C. Huisman, M. D. Hazenberg, J. J. de Soet, J. de Lange, F. R. Rozema

**Affiliations:** ^1^Department of Dental Medical Interaction, Academic Centre for Dentistry Amsterdam, University of Amsterdam and VU University, Gustav Mahlerlaan 3004, 1081 LA Amsterdam, The Netherlands; ^2^Department of Oral- and Maxillofacial Surgery, Academic Medical Center, University of Amsterdam, Meibergdreef 9, 1105 AZ Amsterdam, The Netherlands; ^3^Department of Periodontology, Academic Centre for Dentistry Amsterdam, University of Amsterdam and VU University, Gustav Mahlerlaan 3004, 1081 LA Amsterdam, The Netherlands; ^4^Department of Haemato-Oncology, University Hospital and Charles University Faculty of Medicine Plzen, Alej Svobody 80, 304 60 Pilsen, Czech Republic; ^5^Samuel Oschin Comprehensive Cancer Institute, Cedars-Sinai Medical Center, Los Angeles, CA, USA; ^6^Department of Otolaryngology and Head and Neck Surgery, City of Hope National Medical Center, City of Hope, 1500 East Duarte Road, Duarte, CA, USA; ^7^Department of Hematology, Academic Medical Center, University of Amsterdam, Meibergdreef 9, 1105 AZ Amsterdam, The Netherlands; ^8^Department of Preventive Dentistry, Academic Centre for Dentistry Amsterdam, University of Amsterdam and VU University, Gustav Mahlerlaan 3004, 1081 LA Amsterdam, The Netherlands; ^9^Department of Oral- and Maxillofacial Surgery, VU University Medical Center, VU University, De Boelelaan 1117, 1081 HZ Amsterdam, The Netherlands

## Abstract

Hematopoietic stem cell transplantation (HSCT) is widely used as a potentially curative treatment for patients with various hematological malignancies, bone marrow failure syndromes, and congenital immune deficiencies. The prevalence of oral complications in both autologous and allogeneic HSCT recipients remains high, despite advances in transplant medicine and in supportive care. Frequently encountered oral complications include mucositis, infections, oral dryness, taste changes, and graft versus host disease in allogeneic HSCT. Oral complications are associated with substantial morbidity and in some cases with increased mortality and may significantly affect quality of life, even many years after HSCT. Inflammatory processes are key in the pathobiology of most oral complications in HSCT recipients. This review article will discuss frequently encountered oral complications associated with HSCT focusing on the inflammatory pathways and inflammatory mediators involved in their pathogenesis.

## 1. Introduction

Hematopoietic stem cell transplantation (HSCT) is a potentially curative treatment for patients with various hematological malignancies, bone marrow failure syndromes, and congenital immune deficiencies. Stem cells can be obtained from bone marrow, peripheral blood, or umbilical cord blood. Autologous HSCT (in which stem cells are derived from the patient) is utilized to treat chemosensitive malignancies, such as multiple myeloma, non-Hodgkin, and Hodgkin lymphoma. Its anticancer effect is entirely derived from the high-dose, myeloablative conditioning regimen, whereas a subsequent autologous stem cell infusion enables bone marrow recovery. Allogeneic HSCT (in which the stem cells originate from a related or unrelated donor) is often the preferred treatment in a number of other hematological malignancies such as acute and chronic leukemia and relapsed lymphoma, because of its graft versus leukemia/lymphoma (GvL) effect, which is an immunological response of donor-derived immune cells against malignant cells. In the late 1990s, a better understanding of GvL biology led to preparative regimens that involve less intensive conditioning radio-chemotherapy and are thus less directly toxic than myeloablative regimens. Unlike traditional myeloablative conditioning, these reduced intensity conditioning (RIC) regimens are primarily immunosuppressive to enable engraftment of the transplanted donor cells and depend on the graft to eradicate cancer. RIC transplants can also be conducted in patients previously not eligible for myeloablative protocols, because of older age or medical condition [1]. GvL responses are often accompanied by graft versus host disease (GvHD), a complication of allogeneic HSCT in which donor-derived immune cells including T-, B-, and Natural Killer (NK) cells raise an immune response against normal host tissue, such as the oropharynx, gut, skin, eyes, and liver.

The overall prevalence of oral complications in patients receiving HSCT is estimated to be 80% [2]. Frequently encountered acute oral complications include mucositis, local and systemic infections, oral dryness, and taste changes [3–6]. Whereas in autologous HSCT most of these problems have resolved after 6 months, patients that have been treated with allogeneic HSCT may also later on experience complications associated with GvHD.

Inflammatory processes are the key in the pathobiology of most oral complications in HSCT recipients. This review article will discuss frequently encountered oral complications associated with HSCT focusing on the inflammatory pathways and inflammatory mediators involved in their pathogenesis.

## 2. Oral Mucositis

Oral mucositis (OM) is an inflammatory-driven process of the oral mucosa and is one of the best-studied oral side effects of cancer therapy. It is induced by radiation therapy and/or chemotherapy and is characterized clinically by mucosal damage ranging from mild inflammation presenting as erythematous atrophic lesions to extensive ulcerations penetrating the submucosa. In HSCT recipients, mucositis is not limited to the oral cavity but may occur along the entire orodigestive tract. The mechanisms underpinning the pathobiology of mucositis are thought to be largely the same regardless of the location along this tract.

The incidence of OM has been estimated to range from 75% to 100% following myeloablative conditioning regimens [7] and has been reported as the most painful and debilitating oral complication, significantly impairing quality of life (QoL) [8]. Prospective studies reported that conditioning regimens containing high-dose melphalan, busulphan, and cyclophosphamide in combination with total body irradiation (TBI) were associated with severe OM [9–11]. While OM risk among patients receiving conditioning regimens including TBI exceeds 90%, the risk drops to 30%–50% for individuals being treated with protocols without TBI [12]. Conditioning regimens are the most important parameters determining OM risk, but patient-related factors are also involved, although the association is less clear. In particular the local tissue environment and mucosal responses to damaging stimuli, which may in part be genetically determined, govern the risk, course, and severity of mucosal injury [12, 13]. Genetic determinants of OM risk include genes that regulate the availability of active chemotherapy drug metabolites. For example, evaluation of genetic variation in folate-metabolizing enzymes may help to identify patients at greater risk for methotrexate toxicity, but enzyme deficiencies may be relatively rare [11]. In contrast, differences in the expression of genes associated with biological pathways that drive mucositis are more common. For instance, genetic polymorphisms associated with the expression of inflammatory mediators such as tumor necrosis factor- (TNF-) *α* have been implicated in OM risk in patients undergoing allogeneic HSCT [14]. Recently, Sonis et al. identified a single-nucleotide polymorphisms- (SNP-) based Bayesian network developed from saliva-sourced DNA that may predict an individual's risk to develop severe OM following conditioning for autologous HSCT [12].

OM following RIC regimens is usually less severe and of shorter duration [15, 16]. However, RIC regimens may vary considerably in intensity and accompanying toxicity and more prospective studies are necessary.

Considerable progress has been made in the past years in understanding the pathobiology of mucositis [17–19] and we will summarize recent insights.

### 2.1. Pathobiology of Mucositis

**Figure 1:**
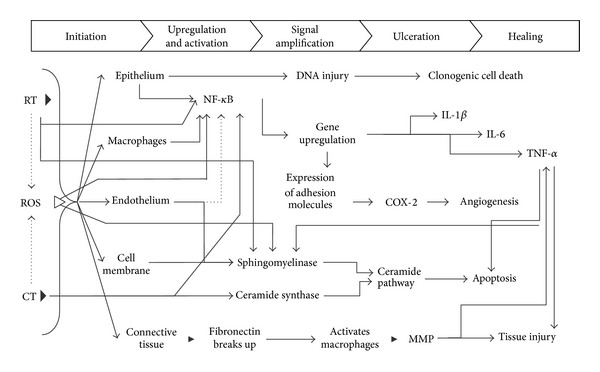
An overview of the pathogenesis of oral mucositis. RT: radiotherapy, ROS: reactive oxygen species, CT: chemotherapy, NF-*κ*B: nuclear factor-kappa B, IL: interleukin, TNF-*α*: tumor necrosis factor-alpha, COX-2: cyclooxygenase-2, and MMP: matrix metalloproteinase. Courtesy of Professor ST Sonis and the Multinational Association of Supportive Care in Cancer (MASCC).

Historically, OM was viewed solely as an epithelium-mediated event that was the result of nonspecific toxic effects of radiation and/or chemotherapy on rapidly proliferating basal epithelial cells resulting in clonogenic cell death. This continues to be a component of a much more complex contemporary model of mucositis developed by Sonis [19]. This five-phase biological model describes a cascade of interrelated and overlapping genetic and histopathological events. These phases include initiation, upregulation/activation, signal amplification, ulceration, and healing involving epithelial and connective tissues of the mucosa (Figure 1).

The initiation phase in the pathobiology of mucositis is characterized by radio- and/or chemotherapy-induced DNA and non-DNA damage that results in injury of basal epithelial cells, submucosal cells, and endothelial cells. In particular submucosal cell death contributes to injury [20]. In response to this damage, reactive oxygen species (ROS) are generated, which amplify DNA damage and clonogenic cell death and activate in the subsequent phase a number of transcription factors, including nuclear factor- (NF-) *κ*B [7, 21].

NF-*κ*B is considered as the “gatekeeper” for various inflammatory pathways involved in mucositis [18]. Activation of NF-*κ*B occurs in virtually all epithelial and submucosal cells and induces expression of adhesion molecules and activation of the mitogen-activated protein kinase (MAPK) and cyclooxygenase- (COX-) 2 pathways [19, 21, 22]. Furthermore, the finding that NF-*κ*B activation can have both proapoptotic and antiapoptotic effects (through apoptosis regulator BcL-2 genes) makes it a significant factor in determining the fate of normal tissues following cytotoxic therapy [22].

Upregulation of NF-*κ*B generates the formation of interleukin- (IL-) 1*β*, IL-6, and TNF-*α* [23]. These proinflammatory mediators stimulate additional injury through positive feedback loops (signal amplification phase), whereby TNF-*α* acts on pathways to reinforce NF-*κ*B activation. Additional support for the role of cytokines has been provided by therapies that interfere with the development of mucositis by influencing the cytokine profile [24–31].

Together with alternative, NF-*κ*B independent pathways such as the ceramide pathway, this results in apoptosis of submucosal and basal epithelial cells leading to mucosal ulceration.

Furthermore, metalloproteinases (MMPs) are involved in the pathobiology of mucositis [32, 33]. Damage to the submucosa is, besides from being a direct effect of radiation or chemotherapy, mediated by the activation of Activating Protein-1 (AP-1), which stimulates the secretion of MMPs by fibroblasts [19, 34]. TNF-*α* is an important regulator of the transcriptional activity of AP-1, by initiating the MAPK signaling pathway activating c-JUN amino-terminal kinase [32]. Moreover, IL-1*β* and COX-2 may induce MMP activation [19]. Increased levels of MMP-2, -3, -9, and -12 are associated with inflammatory infiltrates and maximum tissue damage. In contrast, MMP-1 expression correlates with tissue restitution [32]. No significant correlation was found between levels of MMP-1, MMP-8 (neutrophil collagenase), and MMP-13 (involved in degradation of extracellular matrix (ECM) and bone) in oral rinsing samples and OM scores in allogeneic HSCT recipients [35].

The ulcerative phase comprises loss of mucosal integrity and microbiological colonization with subsequent further proinflammatory cytokine production.

Healing of the oral mucosa has received limited study. It is associated with epithelial proliferation, often concurrent with hematopoietic recovery, reestablishment of local microbial flora, and absence of factors that interfere with wound healing such as infection and mechanical irritation [36]. The ECM plays a significant role in signaling between tissues and is a complex structural network of fibrous proteins, proteoglycans, and glycoproteins. ECM stimulates epithelial cell migration, proliferation, and differentiation, leading to renewal of the mucosa [19]. Epidermal growth factor (EGF), transforming growth factor- (TGF-) *α*, IL-1, and interferon- (IFN-) *γ* appear to promote this process by upregulating TGF-*β*, which stimulate expression of fibronectin and collagen type IV [37]. After healing, the oral mucosa appears normal, but appearances may be deceptive as ongoing angiogenesis and connective tissue maturation result in increased risk for OM if additional cytotoxic therapy is administered [7].

### 2.2. Modifying Factors

Several anti-inflammatory cytokines and growth factors have been identified to have a protective effect. De Koning et al. [38] suggested a protective role of IL-10, after observing more severe intestinal damage following methotrexate treatment in IL-10 deficient mice compared to wild-type controls. Several animal studies indicated that IL-11, an inhibitor and downregulator of inflammatory mediators including nitric oxide (NO), reduces OM [39]. Moreover, it was suggested that subcutaneous administration of IL-11 reduced the severity of OM by maintaining keratin production in epithelial cells, as well as by reducing mucosal proinflammatory cytokine expression [23]. Unfortunately, IL-11 administration caused severe fluid retention and early mortality in a clinical trial, leading to early closure of the study [40]. Topical TGF-*β*3 prior to chemotherapy administration negatively regulated epithelial cell proliferation and reduced OM in a hamster model [41]. Keratinocyte growth factor-1 (KGF-1) has pleiotropic activity. It is mitogenic and promotes cell survival by upregulating BcL-2 genes, which suppress apoptosis [42]. KGF-1 also activates nuclear factor erythroid 2-related factor 2 (Nrf2) that coordinates the expression of cytoprotective genes and upregulates IL-13, an anti-inflammatory cytokine that attenuates the effects of TNF-*α* [37]. Human recombinant KGF-1 has been found beneficial for the prevention of OM in patients treated with high-dose CT and TBI followed by autologous HSCT [31, 43].

An important mediator of local inflammatory responses is the oral and intestinal microbiome. It has been demonstrated in mice that perturbations of local immune responses to the microbiota can lead to spontaneous inflammation, and vice versa, and loss of microbial diversity is associated with a proinflammatory state [44, 45]. Shifts in the commensal oral bacterial flora associated with leukemia, neutropenia, direct effects from anticancer therapy, antibiotic use, hyposalivation, and mucosal surface changes are well documented [46, 47]. For example, results from a study in breast cancer patients suggested a shift to a more complex oral bacterial profile following chemotherapy [48], and a recent study using 16S rRNA and 454 pyrosequencing suggested that chemotherapy-induced changes of the bacterial composition were predictive for OM risk [49]. Certain bacteria may be actively involved in oral mucositis. In HSCT recipients, substitution with coagulase-negative staphylococci (CONS) for streptococci was associated with OM [50]. The presence of* Porphyromonas gingivalis*, a strictly anaerobic Gram-negative microorganism associated with periodontitis, was shown to have a positive predictive value for mucosal ulcerations in HSCT patients [51]. It was suggested that* P. gingivalis* plays a role in the initiation of OM by upregulating Toll-Like Receptors (TLRs), which facilitate NF-*κ*B activation.

Salivary defense proteins and peptides are important determinants of the environment of oral tissues. Their effects are synergistic and in many cases reinforced by immune and/or inflammatory reactions of the oral mucosa. Some defense proteins, like salivary immunoglobulins and heat shock proteins, are involved in both innate and acquired immunity. Cationic peptides and other defense proteins (e.g., lysozyme, bactericidal permeability increasing protein (BPI) salivary amylase, cystatins, proline-rich proteins, mucins, peroxidases, and statherin) are primarily involved in innate immunity [52]. However, the role of saliva in the development of OM is presently unclear [53, 54].

### 2.3. Infectious and Inflammatory Complications of Oral Mucositis

Once ulcerations are manifest, they represent a risk for systemic infection with bacteremia, fungemia, fever, and sepsis, particularly with concomitant neutropenia [7, 55–57]. The damaged mucosa may provide a portal of entry for microorganisms and inflammatory products into the bloodstream [58–60]. OM is the most likely origin of bacteremia with oral viridans streptococci most frequently resulting in fever and may lead to acute respiratory distress syndrome and septic shock [61, 62]. Bacteremia may also be caused by coagulase-negative staphylococci (CONS) originating from the oral mucosa [50, 63]. It should be noted, however, that, in only 30% of patients with mucositis and fever, a bacterial cause could be identified. Mucosal barrier injury likely triggers a systemic inflammatory response, and this response by itself may cause neutropenic fever [57]. Infectious and inflammatory complications associated with ulcerative OM may explain the observation reported in a number of studies that OM is associated with an increased risk to early nontumor-related mortality in HSCT recipients [6, 64, 65].

As described below tissue damage associated with mucositis is also thought to be involved in the pathogenesis of GvHD [66, 67]. However, Vokurka et al., [68] found no evidence for this notion in a study in which OM was prevented by oral cryotherapy in allogeneic HSCT recipients treated with high-dose melphalan conditioning regimens [68].

## 3. Graft versus Host Disease

In allogeneic HSCT recipients, GvHD is frequently encountered and remains a major cause of morbidity and mortality. Organs most at risk to be affected by this complex immunologic disorder include the skin, gastrointestinal tract including the oropharynx and liver, and eyes. Billingham [69] described three fundamental elements required for the occurrence of GvHD. (i) The graft must contain immunologically competent cells (T cells); (ii) the recipient must be incapable of rejecting the graft cells; and (iii) the recipient must express tissue antigens that are not present in the donor [66, 70]. The most important genetically defined proteins on host cells to which donor T cells respond are human leukocyte antigens (HLA). The degree of HLA match of donor to patient is the most important risk factor for GvHD. Additional risk factors include minor histocompatibility antigens (mHA), older age of patient, primary disease, graft source, donor parity and sex mismatch, the toxicity of conditioning regimens, and the effectiveness of GvHD prophylaxis [71, 72]. Once established, GvHD is difficult to treat. As GvHD and GvL responses usually come together, a too rigorous GvHD prophylaxis or treatment may carry the cost of increased risk of disease relapse [73]. GvHD can be classified as either acute GvHD (aGvHD) or chronic GvHD (cGvHD), defined by clinical and pathologic features [74, 75].

### 3.1. Acute Oral GvHD

Oral aGvHD is characterized by mucosal erythema and inflammation, atrophy, and ulcerations. It may be also associated with hyperkeratosis and fibrosis. Salivary gland function and taste may be impaired [76].

In rare cases, hyperacute oral aGvHD may develop as early as one to two weeks after HSCT, but the differential diagnosis of aGvHD, OM, and infection can be challenging [77]. Oral aGvHD should be considered when infection has been excluded and ulcerations fail to heal with hematologic recovery 21 to 28 days after allogeneic HSCT.

The exact incidence of oral aGvHD is not known. The condition is potentially underrecognized, but it is estimated that between 35% and 60% of patients with aGvHD have oral manifestations [76]. A pilot study reported that patients undergoing RIC HSCT developed less oral aGvHD than recipients of myeloablative allogeneic HSCT [78]. A recent observational study in allogeneic HSCT following RIC conditioning reported an incidence of oral aGvHD of 7% [79].

### 3.2. Chronic Oral cGvHD

cGvHD occurs in 40–70% of allogeneic HSCT survivors. The most common sites involved at the initial diagnosis of cGvHD are skin (75%), mouth (51%–63%), liver (29%–51%), and eye (22%–33%) [80].

However, the oral cavity may be the principal and sometimes the only site of involvement [77] and can serve as a useful component of cGvHD diagnosis and staging [75]. Oral cGvHD may affect the mucosa and/or the salivary glands and may develop into mucosal sclerosis. Oral cGvHD as well as its management is associated with increased infection risk [81]. Although oral cGvHD is mild in the majority of patients, it should always be considered as clinically significant due to its often prolonged duration. In a subset of patients it is a continuous source of pain, impairing oral function, affecting alimentation and nutritional status, impeding the maintenance of oral health, and reducing quality of life [82, 83]. In a cohort of RIC HSCT recipients, cGvHD-related oral symptoms developed with a median onset of seven months after transplant persisted for a median duration of six months and reoccurred in one-third of affected patients [79].

### 3.3. Oral Mucosal cGvHD

**Figure 2:**
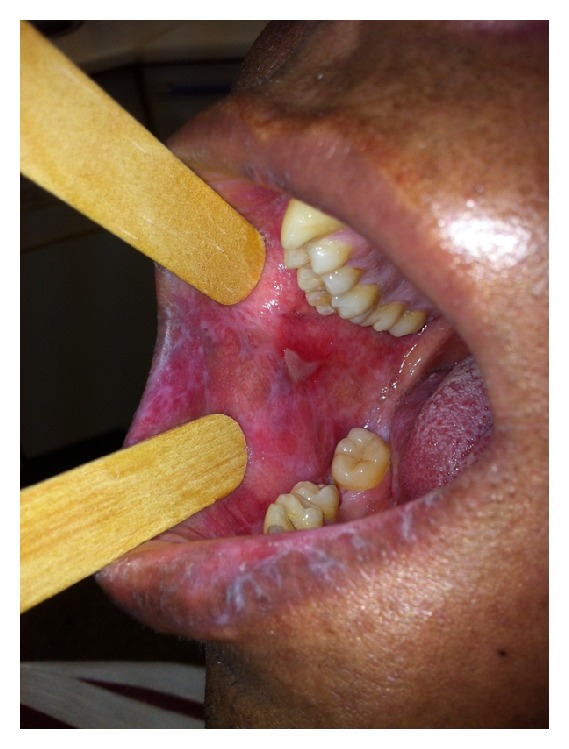
Chronic mucosal GvHD characterized by lichenoid inflammation and pseudomembranous mucositis. Courtesy of Dr. Maria Elvira Correa.

Chronic mucosal GvHD of the oropharynx is estimated to occur in 45–83% of patients [76, 84] and is characterized by lichenoid inflammation particularly affecting the tongue, buccal mucosa, and the lips [85] (Figure 2). Clinical signs resemble those seen in lichen planus and include white hyperkeratotic reticulations and plaques, erythema, and ulcerations, which may be covered with a pseudomembrane. In addition, the gingiva may become atrophic. Early clinical recognition and treatment of GvHD prevent progression into more significant morphological changes [79]. Typical histopathological features include apoptotic bodies, satellite necrosis and lichenoid interface inflammation, and lymphocytic infiltration at the junction of the epithelium and subepithelial connective tissue [74, 79]. Additionally, an inflammatory infiltrate caused by superimposed infection may be present. It is important to be aware of other complications that can mimic oral cGvHD, including oral mucosal reactions to medications (including mammalian target of rapamycin (mTOR) inhibitors), local allergic reactions, infections, and second primary tumors.

### 3.4. Salivary Gland cGvHD

Salivary gland dysfunction is common in HSCT patients and is related to the conditioning regimen, dehydration, medication, and cGvHD.

The primary symptom of salivary gland cGvHD is xerostomia (subjective complaint of oral dryness), but patients may also describe oral sensitivity and burning [81]. cGvHD of the salivary glands is probably underdiagnosed. Some patients experience xerostomia induced by HSCT conditioning (particularly by TBI-containing regimens) and anticholinergic medications, and this may persist through the period when salivary gland cGvHD develops, making onset and diagnosis less evident [86]. Salivary gland cGvHD mimics Sjögren's syndrome, which is commonly associated with xerophthalmia and may also relate to pulmonary GvHD involvement [86]. Saliva plays a critical role in mastication and swallowing, taste, speech, tooth remineralization, the maintenance of oral pH balance, and prevention of oral infections [87].

Patients with salivary gland cGvHD are at risk of developing complications because of diminished salivary defense mechanisms, including antifungal and anticariogenic activities [88, 89]. Castellarin and coworkers reported cGvHD-associated rapidly progressive dental caries with cervical and interproximal involvement [90]. Measurement of resting and stimulated whole-saliva flow rates, individual analysis of the risk of oral diseases, and preventive measures should be part of supportive care in these patients [89].

Another complication of salivary gland cGvHD is the formation of mucoceles. Mucoceles are subepithelial extravasations of sialomucin that occur at the epithelial-connective tissue interface around the obstructed duct of minor salivary glands [74]. Clinically it presents as a soft, fluid-filled elevation of the epithelium [75, 91].

### 3.5. Sclerotic Alterations

Oral sclerotic involvement resulting in limited jaw opening can be the result of perioral and facial skin sclerosis, typically as an extension of generalized sclerotic changes. Facial changes associated with Cushing's syndrome may contribute to reduced jaw opening. Mucosal sclerosis is rare but may develop as a complication of long-standing severe ulcerative mucosal cGvHD [81]. It may restrict jaw opening and tongue movement and may extend to the throat and esophagus resulting in dysphagia. It can be associated with pain and secondary ulceration, significantly impairing alimentation and performing oral hygiene measures [81].

### 3.6. Pathophysiology of Acute and Chronic Oral GvHD

Development of aGvHD occurs in three overlapping and interrelated steps: (i) conditioning; (ii) activation and expansion of alloreactive cells; and (iii) the effector phase [74, 92].

First, conditioning regimens induce tissue damage (e.g., mucositis and skin damage), inducing the release of adenosine triphosphate (ATP) and other damage signals leading to secretion of TNF-*α*, IL-1, IL-6, chemokines, and adhesion factors [93]. This will promote activation and proliferation of host antigen presenting cells (APCs), increased expression of costimulatory molecules, and migration of APCs to secondary lymphoid organs. The impact of the microbiota on GvHD has been recognized to be significant as it has been postulated that the intestinal microflora and endotoxin exert a crucial step in APC activation [94]. Translocation of bacteria and bacterial components through damaged epithelial barriers triggers additional production of inflammatory cytokines, particularly TNF-*α*, IL-1, and IL-12 [66]. A strong correlation between increased TNF-*α* serum levels and aGvHD has been described [95–98]. In reverse, inflammation secondary to GvHD was reported to be associated with major shifts in the composition of the intestinal microbiota, which may aggravate the severity of inflammation [99]. Whether interactions between the oral microbiota and host defense mechanisms may contribute to the pathobiology of oral GvHD remains to be assessed.

During the second phase activated host APCs encounter resting donor T cells and present host antigens to these cells, which become activated, proliferate, and express effector cytokines [100]. Following activation, T cells exit the lymphoid organs, enter the blood circulation, and subsequently migrate to the host target tissues (e.g., skin, orodigestive tract, and liver) [92].

In the third (effector) phase, a complex cascade of cellular mediators and inflammatory agents induce and amplify tissue damage [66, 70]. Donor T cells cause tissue damage via direct cytotoxicity against epithelial cells and release of interferon- (IFN-) *γ* and IL-2 [74]. This activates NK cells and resident macrophages that release proinflammatory cytokines including TNF-*α*, IL-1, and IL-6 leading to amplification of the proinflammatory cytokine cascade (“cytokine storm”) that is a hallmark of acute GvHD [101–103].

A recent study presented an association between IL1B polymorphisms and aGvHD, as well as between IL-1*β* levels, in both saliva and blood, and aGvHD development. In addition, an association was found between the CC genotype and high levels of IL-1*β* suggesting that assessing the kinetics of IL-1*β* in both fluid types may be useful for monitoring the progression of the disease [104].

Selected cytokines involved in GvHD are summarized in Table 1.

CD4+ cells effector function is primarily mediated through secretion of cytokines. Depending on the conditions of activation and subsequent cytokine profile, CD4+ cells can be subdivided into Th1, Th2, Th17, and other subtypes [126]. Although oversimplified, Th1, Th2, and Th17 can be characterized by secretion of IFN-*γ*, IL-4, and IL-17, respectively [127]. Activation of CD8+ cytotoxic T cells is accompanied by increased production of effector molecules, which perpetuate the damage. Both Fas/FasL-dependent and perforin/granzyme-dependent apoptosis are important in GvHD-induced tissue damage [74]. Traditionally, aGvHD is considered to be driven by Th1 cytokines and mediated by CD8+ effectors [70]. However, the pathobiology appears far more complex and B cells may also play a role in the development of aGvHD [128].

Our understanding of cGvHD is limited, partly because of the difficulty with generating animal models that are true representatives of clinical disease. Two theories evolved regarding the pathogenesis of cGvHD. The first theory is end-stage alloreactivity, in which donor T cells augment a Th2 immune response [129]. The second theory postulates that cGvHD is due to poor immunologic recovery with the development of autoreactive T lymphocytes due to lack of thymic control or peripheral mechanisms of deletion [70].

Likely, the pathogenesis of cGvHD begins with uncontrolled expansion of donor T cells in response to both allo- and autoantigens. The activated T cells will subsequently cause target organ damage by inflammatory cytokines, cytolytic attack, and fibrosis and/or by promoting B-cell activation and production of autoantibodies [130]. Evidence exists that B-cell deregulation and the expansion of host B cells [131] contribute to cGvHD. Miklos et al. [132] demonstrated that the presence of antibodies directed to H-Y proteins (a transplantation antigen that can lead to rejection of male grafts by female recipients) correlates with cGvHD. Also, elevated levels of B-cell activating factor (BAFF) contribute to B-cell activation in patients with active cGvHD [133]. Furthermore, Young et al. [134] described that donor B cells are activated by donor CD4+ T cells to upregulate MHC II and costimulatory molecules. Acting as efficient APCs, activated donor B cells enhance donor CD4+ T clonal expansion, thereby augmenting the capacity of these cells to induce autoimmune-like cGvHD.

Indeed, comparable to autoimmune diseases, both T- and B-cell responses appear to play a role in the pathogenesis of cGvHD, suggesting that this reflects a general loss of tolerance including abnormalities in the function of regulatory (Treg) cells. Tregs limit the ability of the immune system to adapt to an inflammatory environment [135]. Impairment of Tregs is associated with loss of peripheral tolerance and with development of cGvHD [136].

Acute GvHD has long been conceived to be dependent upon type I cytokine-driven CD8 effectors, whereas cGvHD has been associated with type II CD4 T cells and Th2 cytokines (IL-4 and IL-10) are considered to be the predominant cytokines in the pathobiology of cGvHD [137]. However, the mechanisms are far more complex, as both aGvHD and cGvHD are initially characterized by increased Th2 cytokines production [138]. Studies have also shown decreased Th2-type cytokine levels among patients who developed cGvHD compared with those who did not [139, 140]. Taken together, evidence of Th2 involvement in cGvHD is limited and, depending upon the animal model used, all 3 major subtypes of CD4 cells (Th1, Th2, and Th17) have been implicated in cGvHD [141].

Imanguli and coworkers systematically examined oral mucosal biopsies and assessed that the clinical severity of oral cGvHD was correlated with the presence of apoptotic epithelial cells. These cells were often found adjacent to infiltrating effector-memory T cells, expressing markers of cytotoxicity and type I cytokine polarization (T-bet+ T cells) [142]. Accumulation of T-bet+ T-cell effectors was associated with increased proliferation and the expression of the type I chemokine receptor CXCR3. In both infiltrating cells and keratinocytes, increased expression of the CXCR3 ligand MIG (CXCL9) and IL-15, IFN-inducible factors, type I differentiation, and expansion of alloreactive effectors was observed [142]. These findings also challenge the current paradigm of cGvHD as a Th2 driven disorder.

**Figure 3:**
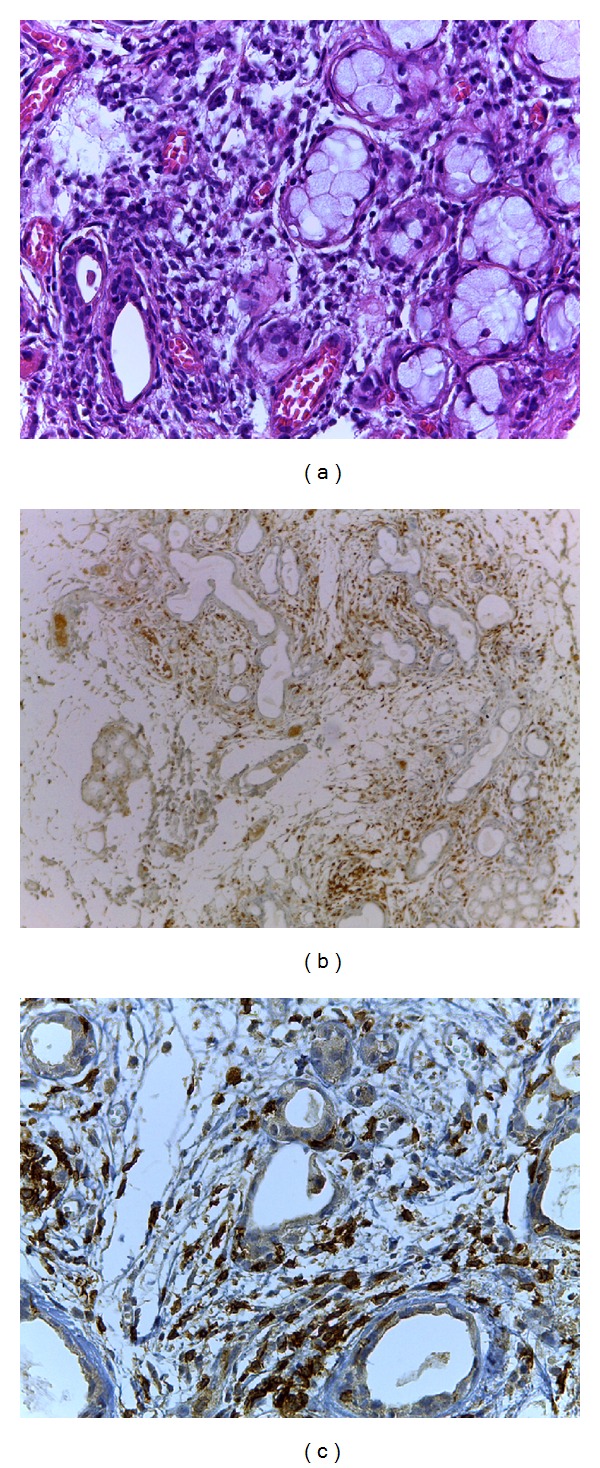
Typical histopathological changes of salivary gland in chronic GvHD. Periductal lymphocytic infiltrate and lymphocyte exocytosis can be noted implying high inflammatory activity. (a) Hematoxylin and eosin stained histological section (original magnification ×400); (b) immunostained section targeting for CD45 (original magnification ×100); (c) immunostained section targeting for CD8. Lymphocytes are shown within ductal epithelium and acinar unit (original magnification ×400). Courtesy of Drs. Tânia C Benetti Soares and Maria Elvira Correa.

Salivary gland cGvHD is characterized by typical histopathological changes (Figure 3) including periductal mononuclear infiltration particularly of CD45, CD45RO, CD4 and CD8 positive cells, atrophy of salivary gland lobules, and periglandular fibrosis [86, 143, 144].

## 4. Infections

Infections, including those from oral sources, are a frequent complication of HSCT [70, 76, 92]. Risk factors for obtaining infections include the underlying malignant disease, the medical condition and comorbidities, the presence of chronic or latent infections, the type of transplant, the source of stem cells, the use of antimicrobials, mucosal barrier loss, immunosuppression and myelosuppression induced by HSCT conditioning, and GvHD and/or GvHD management [145]. Two mechanisms play a major role in infection risk. One depends on nonspecific defenses such as the integrity of surface barriers and presence of systemic or salivary antimicrobial agents, such as defensins [146]. The other major defense against infections is the immune system, of which virtually all components are deficient after HSCT or suppressed by immunosuppressive therapy to prevent GvHD.

Uncomplicated recovery starts with healing of the mucosal tissues and recovery of granulocytes and NK cells about two weeks after myeloablative conditioning. However, T-cell and B-cell immune responses against viral, bacterial, and fungal organisms may be suppressed for a prolonged period of time, particularly in the setting of GvHD. This all results in disturbed homeostasis of the oral cavity and increased infection risk [71].

Oral infections may be associated with a wide variety of microorganisms, including bacteria, fungi, and viruses, virtually all of which may give rise to systemic infectious complications in the transplant population. The time of occurrence and appearance of the lesions may contribute to the differential diagnosis [145]. However, coexistent oral conditions such as OM and GvHD often complicate adequate and prompt diagnosis of opportunistic infections.

### 4.1. Bacterial Infections

As described previously, the oral bacterial flora changes before and following chemotherapy [48]. In a cohort of 37 allogeneic HSCT recipients a significant increase of oral colonization with potentially pathogenic microorganisms (predominantly* Enterococcus faecalis* and* Candida *spp.) was observed over the course of hospitalization [147].

It is important to be aware of modified clinical symptomatology of bacterial infections during the neutropenic phase of HSCT. Erythema, pain, edema, and fever may be the only clinical signs of an otherwise purulent infection.

OM is acknowledged to be the principal risk factor for bacteremia due to oral viridans streptococci (OVS) [148], but bacteremia with CONS may also originate from the oral cavity [63].

In addition to infections related to the oral mucosa, chronic infections associated with the dentition may give rise to complications. These infections typically involve the periapical area, impacted teeth, and the periodontium [149]. Periodontal infections, in particular, may represent an easily overlooked source of bacteremia and systemic infection in neutropenic patients [150, 151]. Periodontal infection and inflammation may contribute to the risk of developing OVS and CONS bacteremia during neutropenia following HSCT [151]. Moreover, the reported positive association between* P. gingivalis* and OM suggests at least a mucosal reaction to this bacterial challenge [51]. There is anecdotal evidence that dental infection and inflammation also contribute to oral GvHD.

### 4.2. Fungal Infections

Candidiasis is typically caused by opportunistic overgrowth of* C. albicans*, a commensal oral yeast. It may be associated with a dry mouth, taste disturbances [152], and mucosal discomfort [153, 154]. Several variables contribute to its clinical expression, including immunosuppression, mucosal injury, and salivary dysfunction. In addition, antibiotics may alter the oral flora, thereby creating a favorable environment for fungal overgrowth [155]. The most common forms of intraoral candidiasis reported in oncology patients are pseudomembranous and erythematous candidiasis [156].

Current prophylactic strategies have reduced systemic candidiasis, although oropharyngeal and esophageal infections remain a common complication with potentially serious consequences.

It has been recognized that different* Candida* species provoke different immunologic reactions, making some strains more virulent than others [157]. During oral infection with* Candida*, a large number of proinflammatory and immunoregulatory cytokines are generated in the oral mucosa [158]. Villar et al. [159] demonstrated that highly invasive strains of* C. albicans* triggered higher levels of proinflammatory cytokines, including IL-1*α*, IL-6, IL-8, and TNF-*α* in epithelial cells and IL-6, IL-8, monocyte chemotactic protein- (MCP-) 1, MCP-2, and granulocyte colony-stimulating factor (GCSF) in endothelial cells.* C. glabrata* was associated with oral ulcerations in HSCT recipients, as described by Laheij et al. [51]. Laheij et al. also identified* C. kefyr* as having a positive predictive value for mucosal ulcerations, but its prevalence is unknown in larger populations. Chen et al. [160] and de Mendonça et al. [161] concluded that* Candida* spp., particularly* C. albicans*, were associated with OM in patients with hematological malignancies. However, Westbrook et al. [162] and Epstein et al. [163] did not report a positive correlation between* Candida* colonization and the presence or severity of OM in HSCT patients. These results indicate that the role of* Candida* species in the pathogenesis of OM remains to be elucidated in more detail.

Interestingly, van der Velden et al. [164] suggested that the mycobiome has a role in the pathogenesis of aGvHD.* Candida* spp. colonization of the mucosa may trigger intestinal aGvHD, potentially by the induction of Th17/IL-23 responses through activation of pattern recognition receptors by fungal motifs. Whether this also may occur in the oral cavity deserves further investigation.

Noncandidal fungal organisms may be also associated with oral infection in immunocompromised cancer patients, including infection by species of* Aspergillus*, mucormycosis, and* Rhizopus* [165]. Lesions associated with these fungi may resemble OM lesions but more detailed microbiologic documentation is needed.

### 4.3. Viral Infections

Viral infections of the oral cavity or the perioral region induce pain and discomfort. This may lead to reduced nutritional intake causing dehydration and malnutrition [166]. Moreover, viruses may give rise to systemic infectious complications and may trigger GvHD [167, 168].

Most often, herpes simplex virus (HSV), varicella-zoster virus (VZV), and Epstein-Barr virus (EBV) infections result from reactivation of latent virus, while cytomegalovirus (CMV) infections can result from either virus reactivation or via a newly acquired virus [169].

Most HSCT recipients receive prophylactic antiviral agents to prevent herpes viral reactivation. This reduces viral shedding and herpetic lesions, although reactivation is still possible in patients receiving prophylaxis [170].

When reactivation of HSV-1 occurs, ulcers may develop and aggravate OM, or they may be confused with this condition [171]. Rüping et al. [172] studied the association of IL-1 and TNF-*α* in oral saliva and viral pathogens with the severity of ulcerations in autologous HSCT. An association of the severity of oral ulceration and HSV reactivation was demonstrated. Similarly van der Beek et al. [173] reported that oral shedding of HSV-1 predicts ulcerations. However, in these studies patients did not receive antiviral prophylaxis, whereas in HSCT recipients getting acyclovir prophylaxis HSV seems not a major etiologic agent of ulcerative OM [174].

VZV infection distributes via dermatomes. Like HSV-1 and -2, VZV is latent in neurons, but viral particles are transported via axons to mucosa where the virus continues replicating in epithelial cells. Increased risk of VZV reactivation extends from three to twelve months after transplant, with allogeneic transplant recipients being at the highest risk [166].

CMV remains a major cause of morbidity, particularly in allogeneic HSCT recipients [175]. Salivary glands seem an important reservoir of CMV [176], as the virus can be shed in saliva and infected saliva is the main mechanism for transmitting CMV [177]. Correia-Silva et al. [176] found a positive correlation between CMV DNA loads in saliva and blood in allogeneic HSCT recipients. Although a recent study did not find a correlation between CMV in oral rinsing samples and oral ulcerations [173], oral ulceration due to CMV and cases of coinfection of HSV and CMV in oral ulcers have been reported [178]. CMV infection causes monocyte and T-cell activation and activation of the TNF system, concomitant with an increase in plasma levels of IL-10 [179]. Moreover, CMV may modulate immunological status of host cells by inducing local production of proinflammatory cytokines such as TNF, IL-1, IL-6, IL-8, and IL-10 [180, 181].

Liu et al. [182] investigated the mechanism of CMV-specific T-cell immune responses after HSCT in CMV-specific CD8+ T cells. They could not define the critical threshold or absolute number of CMV-specific CD8+ T cells that protect against CMV reactivation. Impaired reconstitution of Tregs may also be associated with CMV infection [183].

HSCT recipients may be at risk of developing EBV-related lymphomas and EBV-associated carcinomas of the head and neck region [184]. Posttransplantation lymphoproliferative disorder (PTLD) is the most common malignancy in the first year following allogeneic HSCT. The Waldeyer ring is more frequently involved [185]. Oral involvement of PTLD is rare; it may manifest as a crater-like gingival defect or an ulcerated dark-red mass [186]. In addition, oral hairy leukoplakia has been attributed to EBV infection in HSCT recipients.

Oral lesions caused by nonherpes viruses including adenovirus and human papilloma virus (HPV) have been described. Patients with increased cutaneous HPV lesions will often demonstrate oral lesions. A case of rapidly enlarging, biopsy-documented oral verruca vulgaris in a patient undergoing myeloablative HSCT has been reported [187].

## 5. Taste Dysfunction

Taste dysfunction (dysgeusia) negatively influences QoL and may lead to impaired nutrition and weight loss [188]. While conditioning regimen-related dysgeusia is typically associated with the onset of OM and resolves one to two months following HSCT [189], taste disorders may persist or develop de novo in allogeneic HSCT recipients [190]. Patients may report a rapid decrease in their sense of taste that is temporally associated with the onset or exacerbation of cGvHD, suggesting that the epithelial-derived taste receptor cell is an immune-based target.

Antibiotics may have a negative impact on taste, whereas drugs used to prevent and treat GvHD (e.g., cyclosporine, mTOR inhibitors) can also induce neurological changes that result in altered taste [76].

## 6. Late Complications

With increased numbers of survivors, late effects of HSCT and concomitant therapies have become of increasing importance. Hyposalivation may persist when associated with salivary gland cGvHD, putting patients at risk for rapidly progressing dental demineralization and caries. Other complications include osteoporosis and bisphosphonate-related jaw necrosis. In addition, disturbances in tooth formation and jaw growth and development may be present in pediatric HSCT survivors [191].

Among these late effects, second malignancies have been recognized, including PTLD, hematologic malignancies, and solid tumors [192]. Solid tumors may develop many years after HSCT. In the vast majority of cases, oral tumors are squamous cell carcinomas (SCC). Several authors have described cases of SCC at oral and skin sites previously affected by cGvHD-related inflammatory processes, suggesting that cGvHD is a risk factor. In addition, prolonged immunosuppressive therapy may contribute to SCC risk. In a large retrospective study, HSCT recipients had a 20- to 30-fold risk for oral cancer 10 years or more after allogeneic HSCT [193]. Changes in the oral mucosa due to GvHD can make early detection and diagnosis of SCC clinically challenging. Long-term follow-up of HSCT patients is recommended to detect cancers at an early stage, and patients should be informed of cancer risk and educated to avoid life styles that can potentiate the risk of developing oral SCC.

## 7. Discussion and Suggestions for Future Research

Oral complications contribute significantly to morbidity and sometimes to mortality in HSCT recipients. A better understanding of the pathobiology of these complications and risk factors is necessary in order to develop more efficient preventative and therapeutic strategies.

HSCT conditioning regimens are important parameters determining OM risk, but patients differ considerably in their susceptibility to develop severe OM. Exciting progress has been made in predicting an individual's genetic risk for severe OM following conditioning for autologous HSCT by Sonis et al., who recently identified a SNP-based Bayesian network developed from saliva-sourced DNA [12]. Studies in larger number of HSCT recipients may ultimately result in a clinically useful saliva-based tool predicting severe OM. In addition, studies evaluating the role of the oral microbiome in maintaining oral homeostasis versus aggravating OM are promising [49].

Recently it has been suggested that cancer regimen related adverse events do not occur in isolation but rather develop in clusters [194]. Toxicities may be related with regard to the time of their development only, but there may be also causal relationships in which one toxicity predisposes to a subsequent complication. Moreover, toxicities of cancer treatment may have a common pathobiological background and share genetically determined and other risk factors [195]. As discussed above, inflammation, particularly the upregulation of proinflammatory cytokine pathways, is a major common driver of complications in HSCT recipients and interventions blocking these pathways may affect multiple complications.

Oral complications that seem to be interrelated include OM, hyposalivation, taste alterations, oral infections, and oral GvHD, although in the past most studies have focused on isolated toxicities, and more research is necessary to assess the nature of relationships. Oral complications also link to complications involving nonoral tissues as well as those manifesting systemically. For example, in neutropenic HSCT recipients OM is associated with bacteremia, fever, and sepsis [148]. Such associations may, at least in part, explain the observation that OM is associated with an increased risk to early nontumor related mortality in HSCT recipients [6, 64, 65].

In addition to having a genetic predisposition for (multiple) inflammatory complications of HSCT, the presence of inflammation may lead to a dysregulated and exaggerated inflammatory response following a subsequent inflammatory stimulus. Interestingly, a recent study reported that, at the time of diagnosis of acute leukemia, high prechemotherapy plasma levels of both pro- and anti-inflammatory cytokines and low levels of an antimicrobial protein pro-LL-37 were associated with the highest OM risk, suggesting that a proinflammatory state preceding high-dose chemotherapy may predispose to complications [196].

Similarly, it has been proposed that periodontitis (present before the initiation of cancer treatment) may coinduce an exaggerated inflammatory response following (chemo)radiation in patients with head and neck cancer, leading to more severe OM [197]. It is well established that periodontal disease induces low-grade systemic inflammation characterized by increased levels of proinflammatory cytokines and acute phase proteins in peripheral blood (reviewed by van Dyke and Winkelhoff, 2013 [198]). This is the result of bacteria or bacterial and inflammatory products translocating through inflamed and ulcerated pocket epithelium into the circulation. Periodontitis and OM may be associated with a primed inflammatory response as proposed by the “two-hit” model. The model has previously been used to explain the pathogenesis of acute respiratory distress syndrome (ARDS) following cardiopulmonary bypass [199] and the association between periodontitis and inflammation driven systemic diseases including rheumatoid arthritis [200, 201].

Likewise, it can be hypothesized that oral infections including periodontitis may predispose to OM in HSCT recipients [2]. And taking this concept a step further, it may be speculated that inflammatory conditions (oral or elsewhere) either present before cytotoxic therapy, or developing as a result of therapy may predispose to other inflammation-driven acute or late complications in HSCT recipients. For example, there is anecdotal evidence that periodontal inflammation triggers oral GvHD, whereas GvHD ameliorates when periodontal or other foci of infection are eliminated. Likewise periodontitis and/or OM may be involved in sepsis risk even in the absence of bacteremia. Although a relationship between gastrointestinal mucositis, fever, and increased levels of inflammatory markers in the absence of bacteremia has been proposed [57, 202], the relative contribution of OM to systemic inflammation remains to be investigated.

To explore these hypotheses, longitudinal observational studies involving large numbers of patients looking into associations of oral and nonoral complications and their potentially common underlying mechanisms are needed. Such studies may point to the need of combined risk assessment. Furthermore, investigations using novel techniques will provide an opportunity to evaluate the role of the oral and gastrointestinal microbiome in mucositis and GvHD. Together with studies aimed at assessing the relationship between the microbiome and host factors including epithelial defense mechanisms, this may lead to successful strategies to prevent or ameliorate these complications [203].

Moreover, longitudinal studies using salivary samples may identify genetic risk factors for OM as well as for other regimen related complications [12]. In addition, studies may identify salivary biomarkers that may predict or facilitate diagnosis of OM and GvHD or their response to therapy.

In conclusion, a more holistic approach that includes clinical and translational studies on oral as well as nonoral complications of HSCT may lead to a better understanding of potential commonalities between complications and may open new avenues for prevention and treatment.

## Figures and Tables

**Table 1:** Selected inflammatory cytokines involved in graft versus host disease; intervention in cytokine production is an obvious approach to ameliorate GvHD, but because cytokines have complex and pleomorphic effects, this may result in unintended consequences.

Cytokine	Main role in GvHD	References
TNF-*α*	Activates APCs and enhances alloantigen presentation, recruits effector cells to target organs mediated by inflammatory chemokines and directly causes tissue necrosis.	[66, 105–107]

IL-1*α*	Higher salivary IL-1*α* is associated with oral dryness in oral cGvHD.	[108]

IL-1*β*	Primary activator of chemotactic cytokines and expression of adhesion molecules that facilitate the migration of leucocytes into tissues.	[66, 109]

IL-6	Key factor in CD4+ T cell-dependentaGvHD and inhibition of Tregs. Moreover, an association between IL-6 and the severity of oral cGvHD has been reported.	[108, 110, 111]

IL-2	Critical for T-cell differentiation.	[98]

IL-15	IL-2-like cytokine, enhances T-cell and NK cell proliferation and improves immune reconstitution after allogeneic HSCT.	[98]

IL-12	Pro-inflammatory cytokine involved in the activation of donor T cells. Stimulates T cell and NK cell proliferation and induces maturation of Th1 cells.	[98]

IFN-*γ*	Complex role in innate and adaptive immune responses. IFN-*γ* is able to increase chemokine receptors, MHC proteins and adhesions molecules. Renders monocytes and macrophages more sensitive to LPS stimulation, and amplifies GvHD by direct damage to epithelial cells and by NO-mediated immunosuppression. May just reflect the presence of large numbers of activated T cells, and does not necessarily imply a role of this cytokine in the pathogenesis of GvHD.	[66, 112]

IL-17	IL-17 is mainly produced by activated Th-17 cells, stimulates IL-6 and IL-8 secretion and enhances the expression of adhesion molecules. May play a role in either triggering or aggravating aGvHD and cGvHD, but detailed role remains to be elucidated.	[113–115]

IL-18	Affects both Th1 and Th2 mediated responses and is elevated in aGvHD. In mouse models, administration of IL-18 early after allogeneic HSCT attenuated aGvHD by decreasing Th1 cytokine production.	[98]

IL-5	Produced by T cells, mast cells and eosinophils has been associated with aGvHD. Stimulates B cell growth and increases immunoglobulin secretion; is a key mediator in eosinophil activation.	[98, 116]

IL-10	Inhibits secretion of IL-1, TNF, IL-6, IL-8 and IL-12 from monocytes/macrophages and secretion of IFN-*γ* and IL-2 by T-cells. A protective role for donor-derived IL-10 has been suggested in aGvHD, as it decreases apoptosis. High concentrations of IL-10 in aGvHD may reflect systemic infection and may contribute to immunodeficiency.	[98, 117–120]

IL-21	IL-21 enhances Th1 and Th17 differentiation while inhibiting the conversion of inducible Tregs from naive T cells.	[121]

IL-22	Structurally related to IL-10 and is secreted by Th17 cells and innate immune cells. In line with these findings, IL-22 may act as a protective regulator of tissue sensitivity to GvHD.	[122, 123]

IL-7	Is associated with the development of aGvHD, but mechanism is not well understood.	[98, 124, 125]

GvHD: Graft versus Host Disease, TNF: Tumor Necrosis Factor, APCs: Antigen Presenting Cells, IL: Interleukin, CD4+ T cell: T helper cell expressing surface protein *CD4*, aGvHD: acute GvHD, Treg: regulatory T cell, NK cell: Natural Killer cell, HSCT: Hematopoietic Stem Cell Transplantation, Th cells: T helper cells, IFN-*γ*: *interferon* gamma, MHC: Major Histocompatibility Complex, LPS: lipopolysaccharide, NO: Nitric Oxide, cGvHD: chronic GvHD, GI: Gastro-Intestinal.
